# Noblestitch® system for PFO closure: A novel but judicious alternative to traditional devices—A case report

**DOI:** 10.3389/fcvm.2023.1095661

**Published:** 2023-03-30

**Authors:** Eustaquio Maria Onorato, Luca Grancini, Giovanni Monizzi, Angelo Mastrangelo, Franco Fabbiocchi, Antonio L. Bartorelli

**Affiliations:** ^1^ University Cardiology Department, Cardiologia Universitaria, I.R.C.C.S. Ospedale Galeazzi-Sant'Ambrogio, Milan, Italy; ^2^ Department of Biomedical and Clinical Sciences, “Luigi Sacco”, University of Milan, Milan, Italy

**Keywords:** patent foramen ovale, transcatheter closure, suture-based PFO closure, atrial septal tear, residual shunt

## Abstract

**Background:**

Percutaneous suture-mediated patent foramen ovale (PFO) closure has recently been used with the aim of avoiding double-disc nitinol device implantation. This novel technique has been carried out successfully in several centers offering PFO closure with an effective closure rate comparable to conventional double-disc devices.

**Case summary:**

A 50-year-old man, a pentathlon athlete, suffering from a previous left-sided ischemic stroke, underwent percutaneous closure of a permanent right-to-left shunt *via* PFO with a large fenestrated septum primum aneurysm at another institution. The NobleStitch® system was successfully implanted using local anesthesia and under angiographic-fluoroscopic monitoring. He was discharged home on aspirin 100 mg daily with a moderate residual shunt on contrast transthoracic echocardiography (cTTE) that persisted unaltered at subsequent controls. After 7 months, unable to resume sporting activity because of physical discomfort and dyspnea on exertion, the patient asked for a second opinion at our Heart and Brain clinic. Two-dimensional (2D) TTE showed septum primum laceration next to a radiopaque polypropylene knot with a moderate bidirectional shunt located at the fenestrated septum primum far from the PFO site. A catheter-based closure of the septal defect was therefore planned under local anesthesia and rotational intracardiac echo monitoring. An equally sized discs 28.5 mm × 28.5 mm Flex II UNI occluder (Occlutech GmbH, Jena, Germany) was successfully implanted across the atrial septal defect without complications. The patient was discharged in good clinical conditions; dual antiplatelet therapy (aspirin 100 mg/daily and clopidogrel 75 mg/daily) was recommended for 2 months and then single antiplatelet therapy (aspirin100 mg/daily) up to 6 months. Abolition of the residual shunt was confirmed at 1- and 6-month follow-up by contrast transcranial Doppler and 2D color Doppler cTTE.

**Discussion:**

Closing a PFO with a suture-base system, without leaving a device implant behind, may be a cutting-edge technology and potential alternative to traditional devices. Nevertheless, meticulous selection of the PFO anatomies by 2D TEE is key to a successful closure procedure in order to avoid complications that must be managed again with a second percutaneous procedure or by surgery.

## Introduction

In carefully selected patients with cryptogenic stroke, patent foramen ovale (PFO) closure has been shown to significantly reduce the risk of recurrent neurological events and new brain infarcts compared with antiplatelet treatment alone (1–3).

Conventional PFO nitinol double-disc occluders, routinely used for this purpose, are associated with potential but extremely low risk of early and late complications (arrhythmias, thrombus formation, embolization, erosion). Among the limitations of traditional occluders, future transseptal puncture and left-sided interventions may be impeded by the presence of a bulky device covering the interatrial septum.

Recently, a percutaneous suture-mediated system to close the PFO has been added to interventional practice with encouraging results in terms of safety and efficacy, as long as a meticulous selection of suitable PFO anatomies is carried out. Currently, closure rates with the suture-mediated system appear to be similar to those obtained with double-disc metal-alloy occluders (4). Nevertheless, there is still a paucity of data describing in detail the efficacy and safety of the NobleStitch® system.

## Case presentation

**Figure 1:**
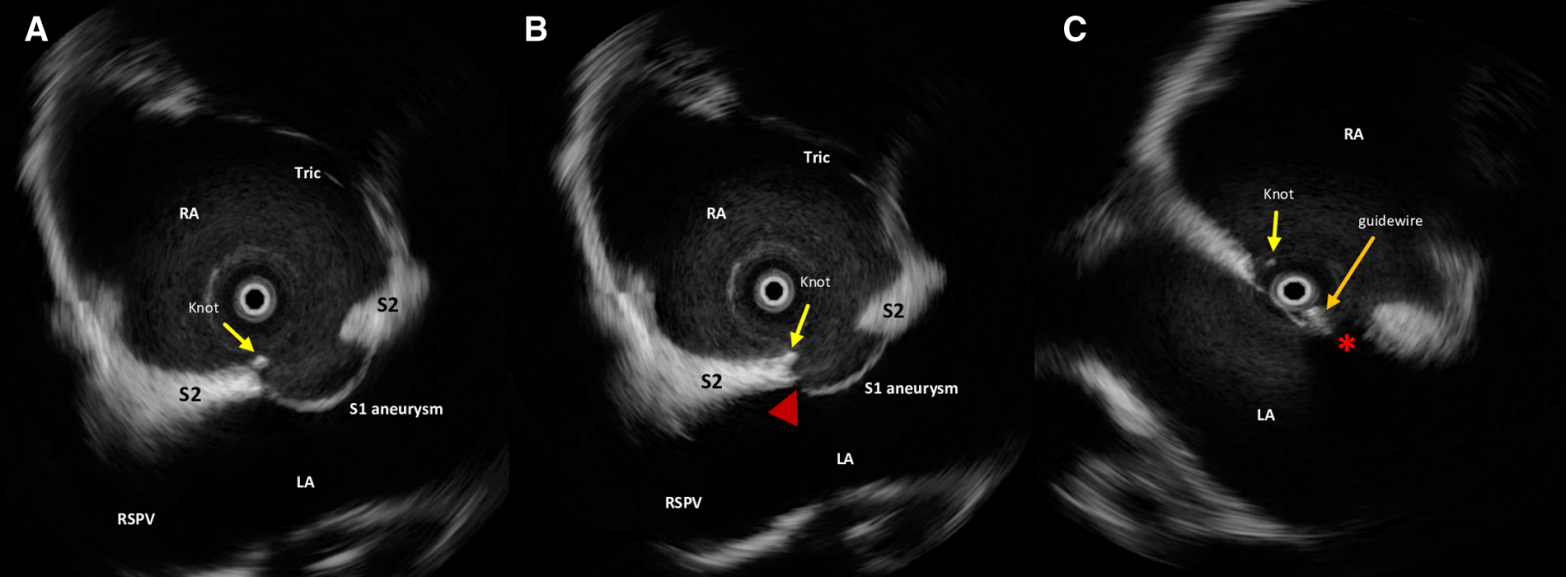
Intraprocedural rotational intracardiac Echo by Ultra ICE (mechanical 9F/9 MHz 360° scan probe, Ultra ICE™, EP technologies, BSC, CA, United States) in the parasagittal long axis (“four chamber”) plane showing septum primum aneurysm, NobleStitch EL sealing system (radiopaque polypropylene knot, yellow arrow), laceration of the septum primum next to the knot (red arrowhead) (**A,B**) and the guidewire (orange arrow) crossing the septal fenestration (red asterisk) (**C**). S1, septum primum; S2, septum secundum; RA, right atrium; LA, left atrium; RSVP, right superior pulmonary vein; Tr, tricuspid valve.

**Figure 2:**
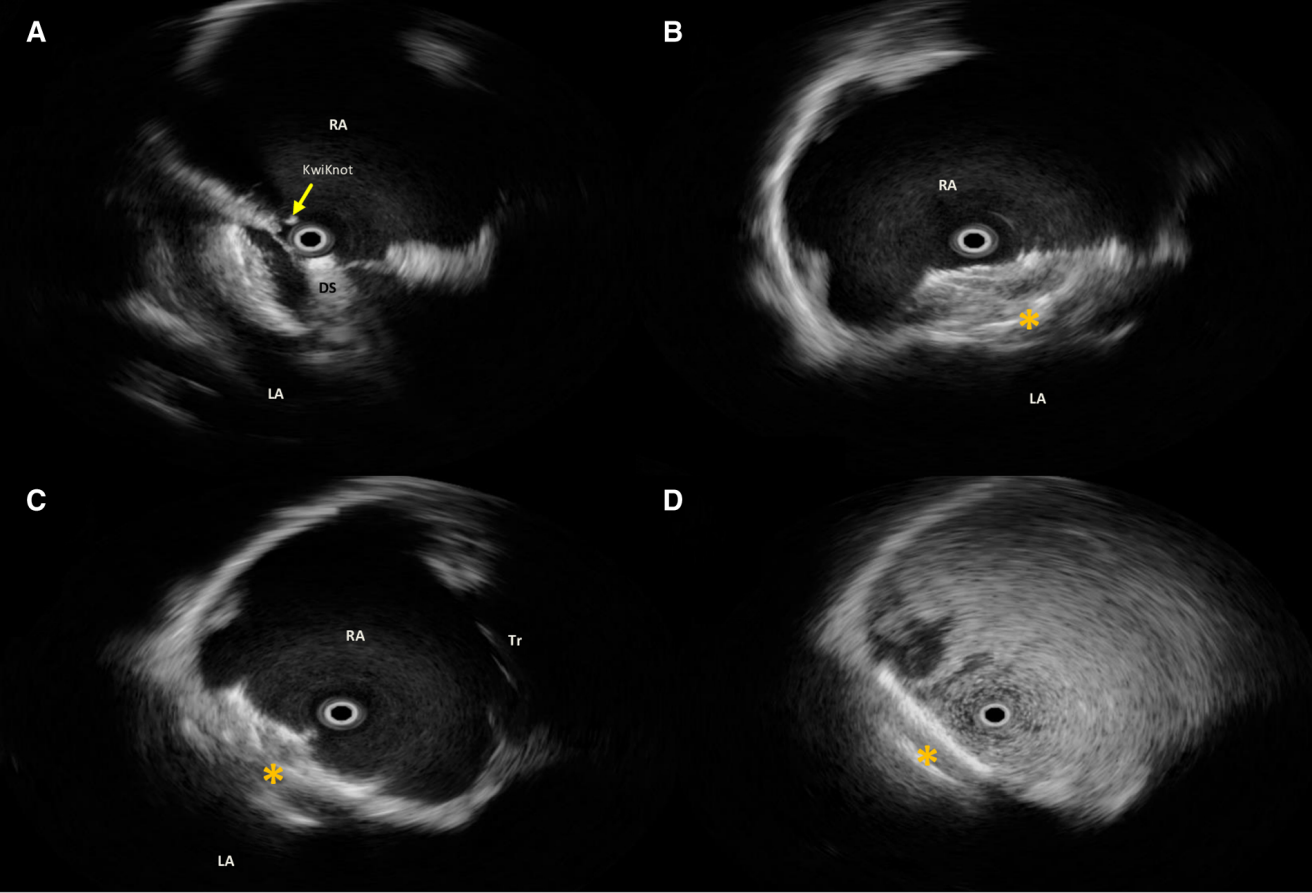
Intraprocedural rotational intracardiac Echo by Ultra ICE (mechanical 9F/9 MHz 360° scan probe, Ultra ICE™, EP technologies, BSC, CA, United States) in the parasagittal long axis (“four chamber”) plane showing procedural steps (**A–C**) of implantation of Flex II UNI occluder (Occlutech GmbH, Jena, Germany) with equally sized discs (28.5 mm × 28.5 mm) (orange asterisk); (**D**) after agitated saline solution injection from the femoral vein, no residual shunt immediately after device release. RA, right atrium; LA, left atrium; DS, delivery sheath across the septal fenestration.

**Figure 3:**
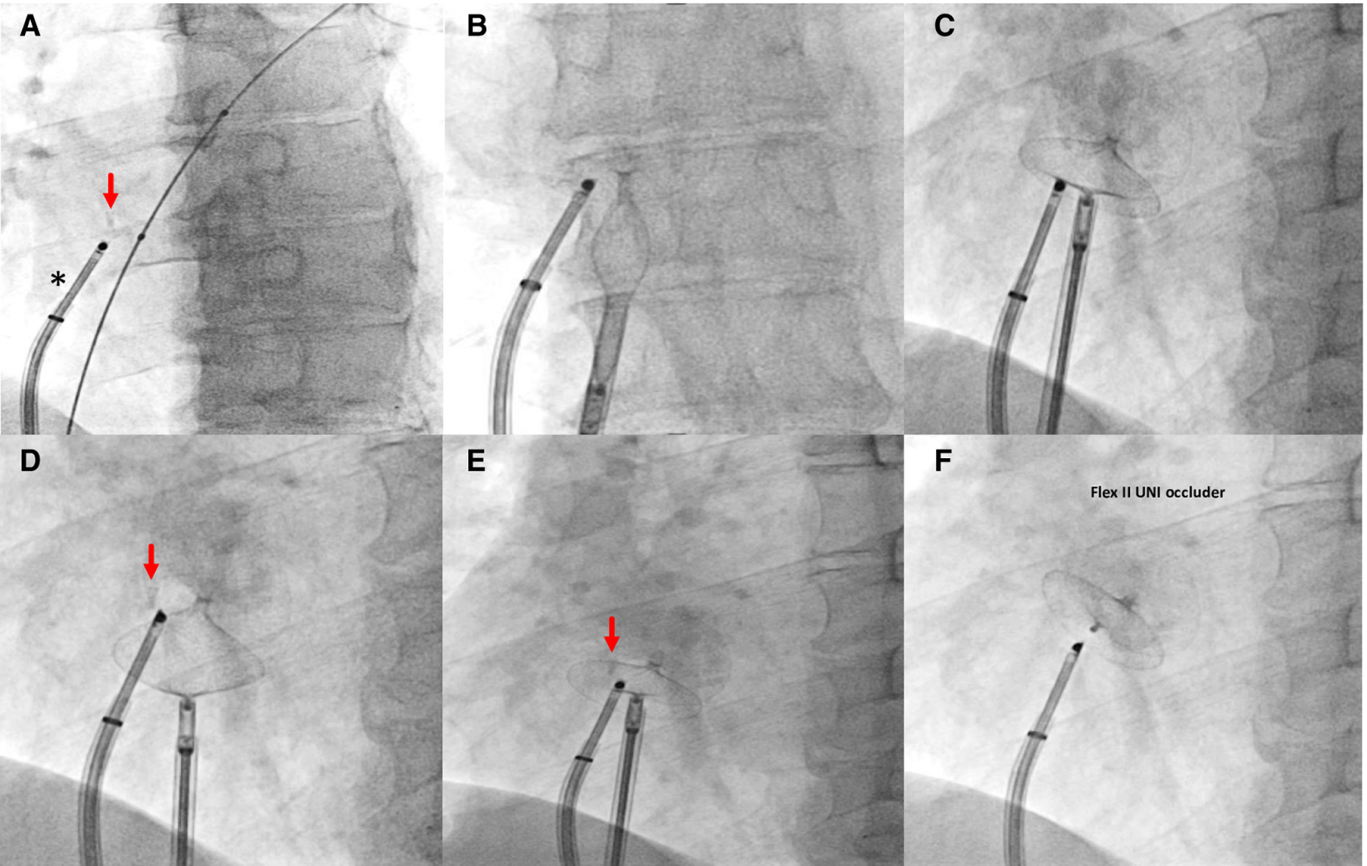
Fluoroscopic steps of implantation of Flex II UNI occluder (Occlutech GmbH, Jena, Germany) with equally sized discs (28.5 mm × 28.5 mm) under rotational intracardiac echo by Ultra ICE (black asterisk). (**A–C**) left and right disc opening; (**D,E**) pull and push maneuver to test device stability; (**F**) device deployed. Red arrow, radiopaque polypropylene knot.

A 50-year-old man, a professional pentathlon athlete without documented risk factors for heart disease, suffered from a left-sided ischemic stroke with dysarthria and right hemianopsia. He was admitted to the stroke unit of another institution where brain magnetic resonance imaging (MRI) showed left posterior insular cortex ischemic stroke. Thrombolysis with intravenous tissue plasminogen activator (IV-tPA) was administered. Afterward, contrast transthoracic and transesophageal echocardiography (cTTE/TEE) and contrast Transcranial Doppler (cTCD) were performed at the Cardiology Department of this institution confirming “spontaneous severe right-to-left shunt (RLS) via a PFO 17.5 mm in length, >5 mm in width associated with abnormally redundant fenestrated septum primum aneurysm (ASA) protruding predominantly to the right atrium in basal conditions and to the left atrium during the cardiorespiratory cycle” (Supplementary Figure S1). Notwithstanding, the PFO anatomy was considered appropriate by the local team, and it was decided to implant a suture-based “deviceless” NobleStitch® EL system, consisting of two polypropylene sutures (one for the septum primum and one for the septum secundum) tightened together by a dedicated delivery and sealing system (KwiKnot). The patient was discharged home on aspirin 100 mg daily for 1 month. Two-dimensional (2D) TTE color Doppler at discharge showed a moderate residual bidirectional shunt, primarily left-to-right across the septum primum fenestration (septal defect), that persisted unaltered on subsequent controls. After 7 months, the patient was unable to resume his sporting activity and attended our Heart and Brain clinic for a second opinion due to tiredness, dyspnea on exertion, and persistent mild dysarthria, a sequela of the index event. Luckily, no new lesions were found at the control brain MRI. Nevertheless, 2D TTE color Doppler confirmed the redundant ASA, the small residual shunt across the tear next to the radiopaque polypropylene knot, and the persistent predominant left-to-right shunt through the atrial septal defect located far from the PFO site. After the heart team discussion, written informed consent was obtained from the patient. A catheter-based atrial septal defect closure was planned under local anesthesia and rotational intracardiac echo (Ultra ICE, Boston Scientific Corporation, San Jose, CA, United States) monitoring, the standard procedural guidance at our center. The tearing of the aneurysmatic septum primum, next to the KwiKnot and probably induced by the stretching of the NobleStitch® device, and the septal defect were clearly visible by rotational intracardiac echo (Figures 1A–C). In order to anchor the device on septum secundum rims, a Flex II UNI occluder (Occlutech GmbH, Jena, Germany) with equally sized discs (28.5 mm × 28.5 mm) was successfully implanted under intracardiac echo (Figures 2A–C) and fluoroscopic guidance (Figures 3A–F) across the septal defect, “sandwiching” the KwiKnot between the two discs and covering the septum primum laceration next to the knot. Intraprocedural agitated saline injection from the femoral vein confirmed abolition of the interatrial residual shunt (Figure 2D). The patient was discharged home the following day in good clinical conditions. Dual antiplatelet therapy (aspirin 100 mg/daily and clopidogrel 75 mg/daily) was recommended for the first 2 months and then single antiplatelet therapy (aspirin 100 mg/daily) up to 6 months.

**Figure 4:**
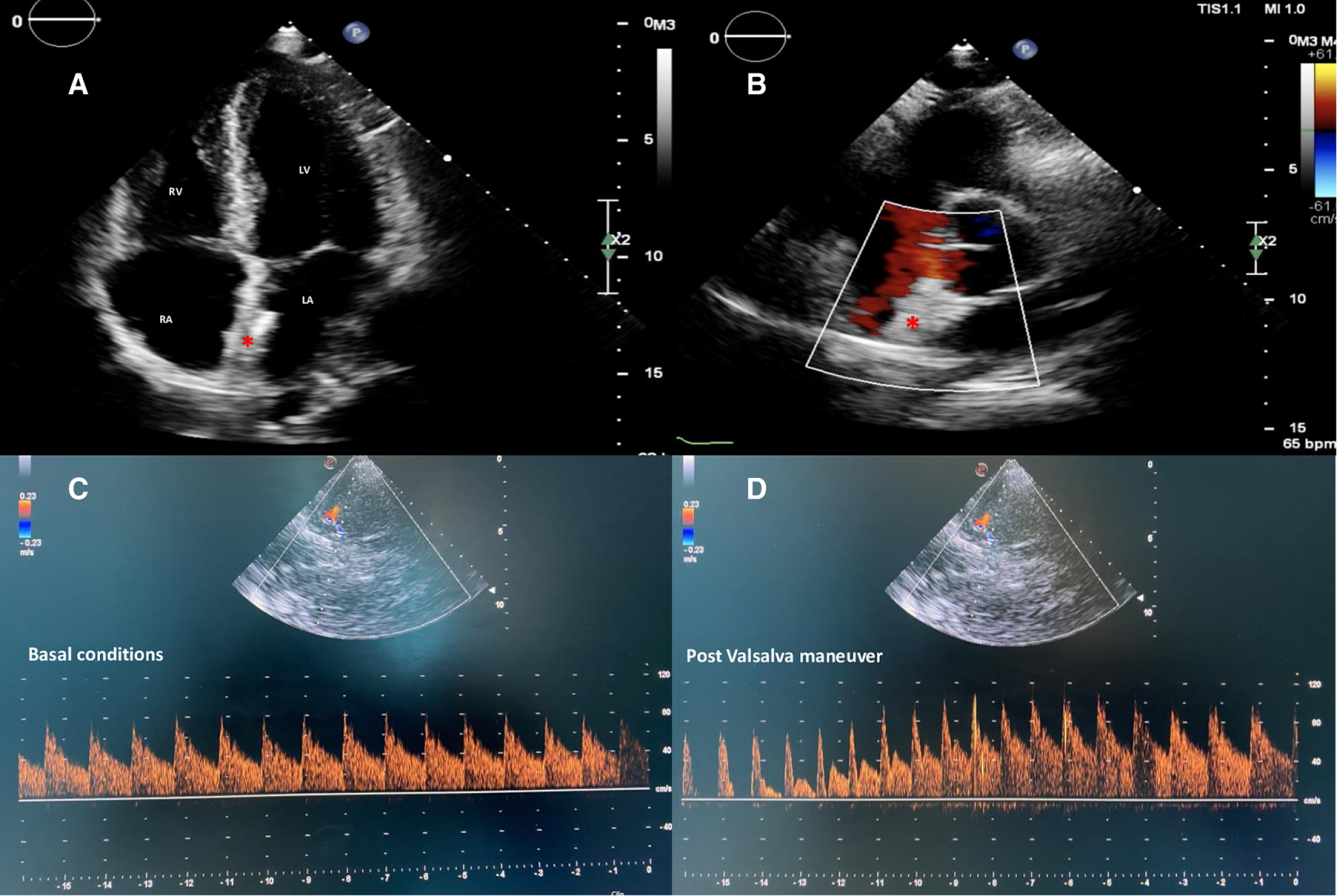
Two-dimensional TTE color Doppler in apical four-chambers (**A**) and parasternal short-axis (**B**) views showing a correct and stable position of the equally sized discs nitinol device (red asterisk) without residual shunt. cTCD confirming abolition of the interatrial shunt both in basal conditions (**C**) and after adequate Valsalva maneuver (**D**). TTE, transthoracic echocardiography; cTCD, contrast transcranial Doppler.

cTTE and cTCD at 1- and 6-month follow-up confirmed the stable position of the nitinol double-disc device without residual shunt (Figure 4). Currently, the dysarthria has improved, and the patient has resumed competitive sporting activity 2 months after the procedure.

## Discussion

The concept of closing a PFO with a suture-based system, without leaving a device implant behind, is very innovative and a potential alternative to nitinol double-disc devices. The NobleStitch® system closes the PFO by applying sutures through the septum primum and septum secundum, subsequently creating a knot between the two sutures, and removing excess suture material. Only one registry has been published (4) with effective PFO closure in the majority of septal anatomies without complications. Thereafter, a retrospective observational study of 247 patients sought to assess PFO anatomy by preprocedural TEE in patients deemed suitable for suture-mediated technique to identify predictors of the most common complication, a post-procedural residual shunt (5). This study revealed that PFO > 5 mm in width and spontaneously large RLS are less likely to be closed with one stitch only. More recently, another prospective single-center study with a 6-month follow-up period (*n* = 116) investigated factors that may have contributed to residual intracardiac shunting ≥2 (20%, *n* = 23), revealing that the principal causes were partial stitch detachment (*n* = 12), atrial septal tear (*n* = 3), and KwiKnot embolization (*n* = 2) (6).

Moreover, a modified technique involving double suture of the septum primum resulted in improved efficacy of the procedure by increasing the surface contact between the septa without the need to place a second septum secundum stitch. However, the current findings are limited by the few cases included in the report (7).

Finally, the suture-based “deviceless” NobleStitch® system has not been gradually growing in importance, and there is limited experience worldwide. On the other hand, an increasing number of reports have documented iatrogenic septum primum lacerations resulting from the use of the NobleStitch® system. These lacerations can cause persistent residual shunts that do not reduce or disappear over time and, as a result, require management, either with a second percutaneous procedure using conventional devices or by surgery (8–10).

Lastly, a PFO Comparative Trial (STITCH) is recruiting patients in the US and European Union with the aim of comparing the effective closure rate of NobleStitch EL vs. FDA Approved Amplatzer Occluder Device (ClinicalTrials.gov identifier: NCT04339699).

It is noteworthy to mention that longer follow-up is imperatively justified to clarify not only the efficacy, but also the safety of this novel but selective alternative to traditional devices.

## Conclusion

If PFO closure to prevent recurrent cryptogenic stroke is here to stay, it is imperative to avoid any residual shunts during mid- and long-term follow-up. While suture-based PFO closure systems offer a promising alternative to conventional occluders, a meticulous patient selection process is essential. Specifically, it is necessary to exclude unsuitable PFO anatomies, such as a prominent and fenestrated ASA, PFO > 5 mm in width, septum secundum lipomatous hypertrophy, and spontaneously large RLS, to achieve procedural success and reduce the risk of new embolic events.

## Data Availability

The original contributions presented in the study are included in the article/**Supplementary Material**, further inquiries can be directed to the corresponding author.
