# Supplementary figures and images for: Noblestitch® system for PFO closure: A novel but judicious alternative to traditional devices—A case report
Source: Front Cardiovasc Med. 2023 Mar 30;10:1095661. doi: 10.3389/fcvm.2023.1095661 (PMC10097917; doi:10.3389/fcvm.2023.1095661)

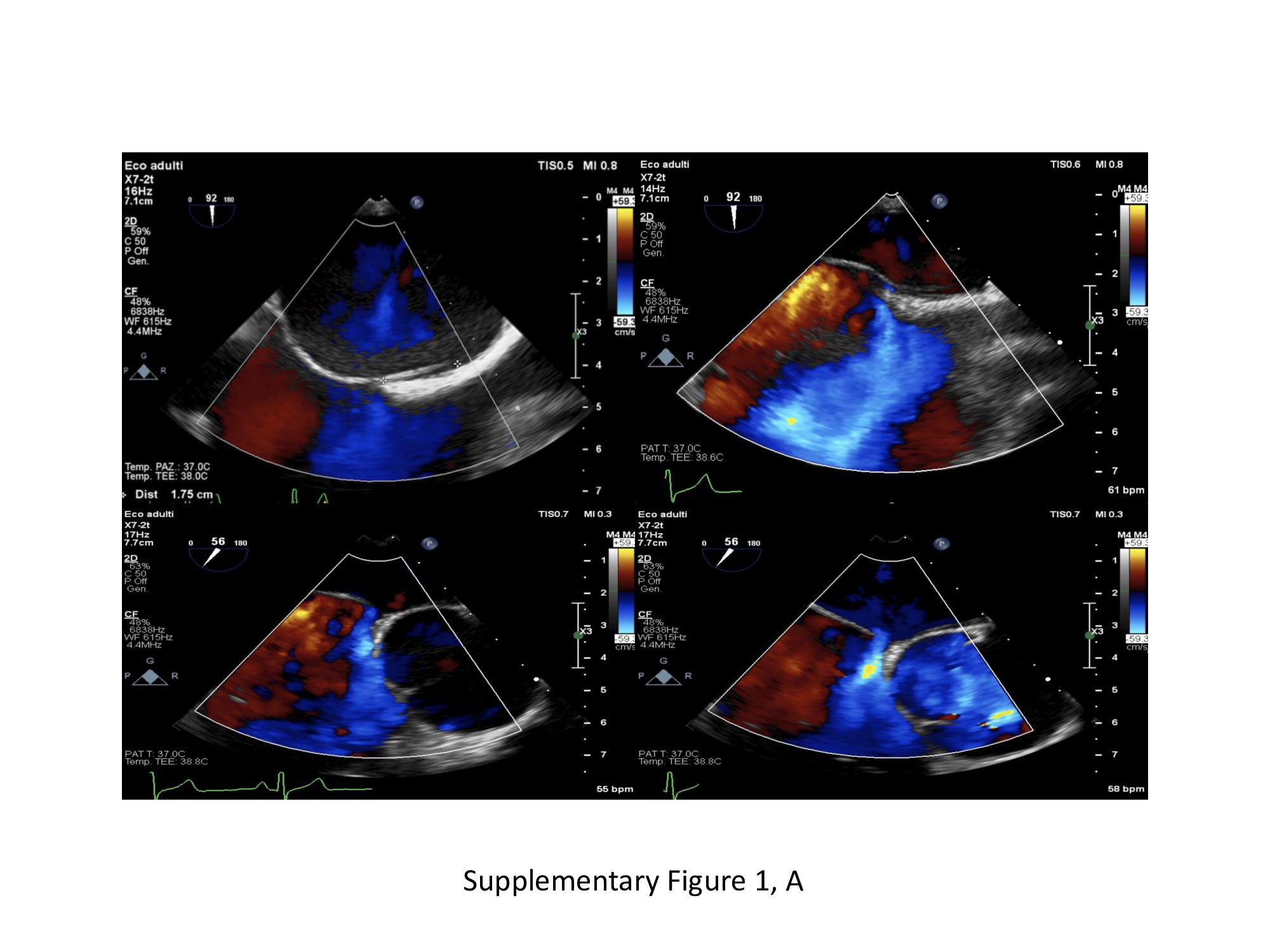

Supplement: Supplementary file 1 [file Image1.jpeg]

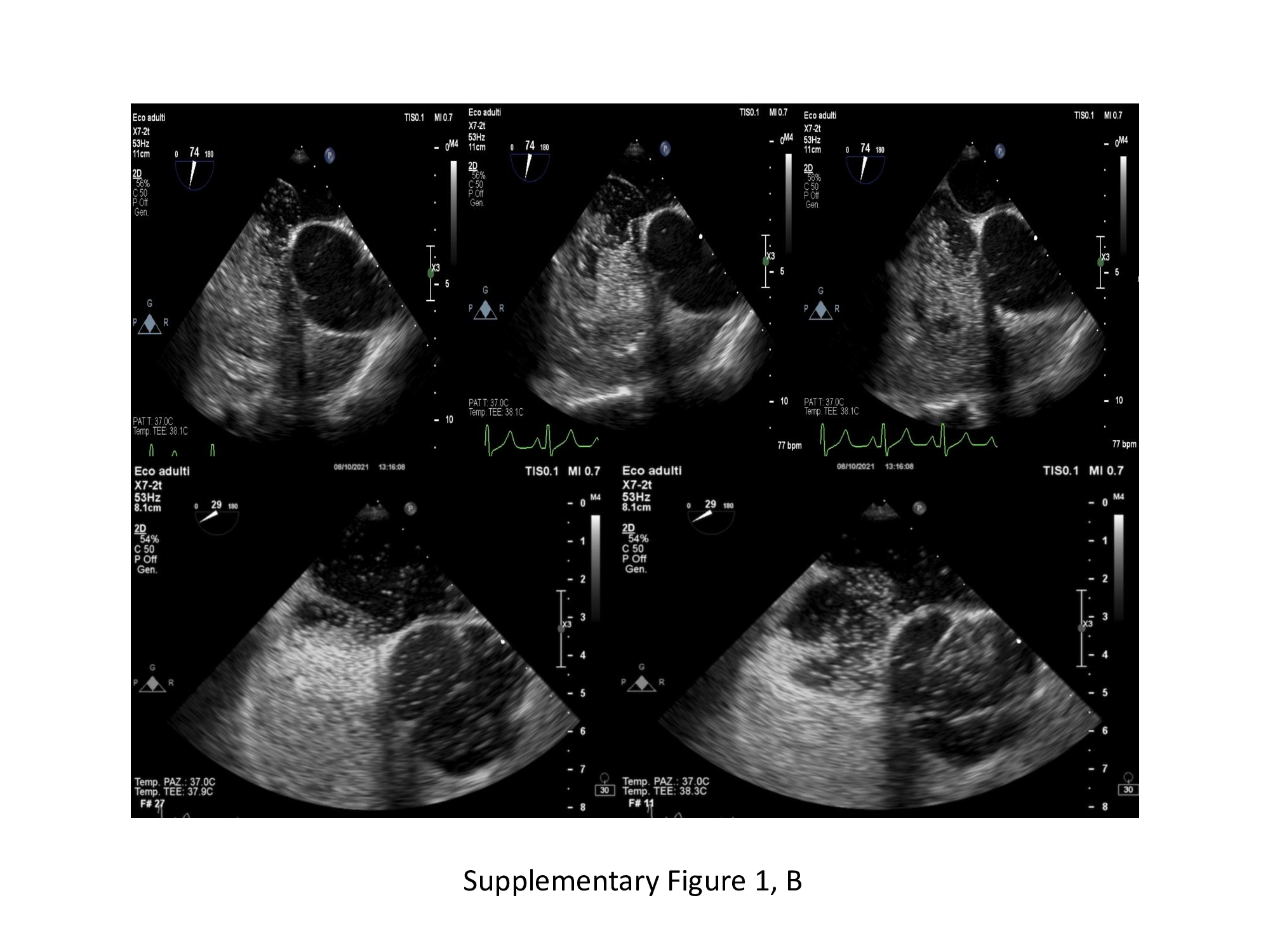

Supplement: Supplementary file 2 [file Image2.jpeg]
